# *In Silico* Discovery of Novel Potent Antioxidants on the Basis of Pulvinic Acid and Coumarine Derivatives and Their Experimental Evaluation

**DOI:** 10.1371/journal.pone.0140602

**Published:** 2015-10-16

**Authors:** Rok Martinčič, Janez Mravljak, Urban Švajger, Andrej Perdih, Marko Anderluh, Marjana Novič

**Affiliations:** 1 Laboratory of Chemometrics, National Institute of Chemistry Slovenia, Hajdrihova 19, 1000, Ljubljana, Slovenia; 2 Faculty of Pharmacy, University of Ljubljana, Aškerčeva 7, 1000, Ljubljana, Slovenia; 3 Blood transfusion centre of Slovenia, Šlajmerjeva 6, 1000, Ljubljana, Slovenia; 4 Laboratory for Biocomputing and Bioinformatics, National Institute of Chemistry Slovenia, Hajdrihova 19, 1000, Ljubljana, Slovenia; University of Parma, ITALY

## Abstract

A pigment from the edible mushroom *Xerocomus badius* norbadione A, which is a natural derivative of pulvinic acid, was found to possess antioxidant properties. Since the pulvinic acid represents a novel antioxidant scaffold, several other derivatives were recently synthetized and evaluated experimentally, along with some structurally related coumarine derivatives. The obtained data formed the basis for the construction of several quantitative structure-activity and pharmacophore models, which were employed in the virtual screening experiments of compound libraries and for the prediction of their antioxidant activity, with the goal of discovering novel compounds possessing antioxidant properties. A final prioritization list of 21 novel compounds alongside 8 established antioxidant compounds was created for their experimental evaluation, consisting of the DPPH assay, 2-deoxyribose assay, β-carotene bleaching assay and the cellular antioxidant activity assay. Ten novel compounds from the tetronic acid and barbituric acid chemical classes displayed promising antioxidant activity in at least one of the used assays, that is comparable to or even better than some standard antioxidants. Compounds **5**, **7** and **9** displayed good activity in all the assays, and were furthermore effective preventers of oxidative stress in human peripheral blood mononuclear cells, which are promising features for the potential therapeutic use of such compounds.

## Introduction

The constant production of free radical species in the body is implicated in the development of several diseases, such as cancer, diabetes, neurodegenerative diseases, cardiovascular diseases, and ageing, but fortunately their harmful effects can be alleviated by endo- and exogenous antioxidants. These are defined as “substances that, when present at low concentrations in comparison to the oxidisable substrate, significantly delay or prevent the oxidation of that substrate” [1]. They are especially important for their ability to prevent/reduce the damage, caused by oxidative stress, which occurs when there is an increased production of oxidizing species in the organism. Several natural antioxidants have been well characterized to date, belonging mostly to the classes of vitamins, carotenoids, polyphenols, and flavonoids [2].

**Fig 1:**
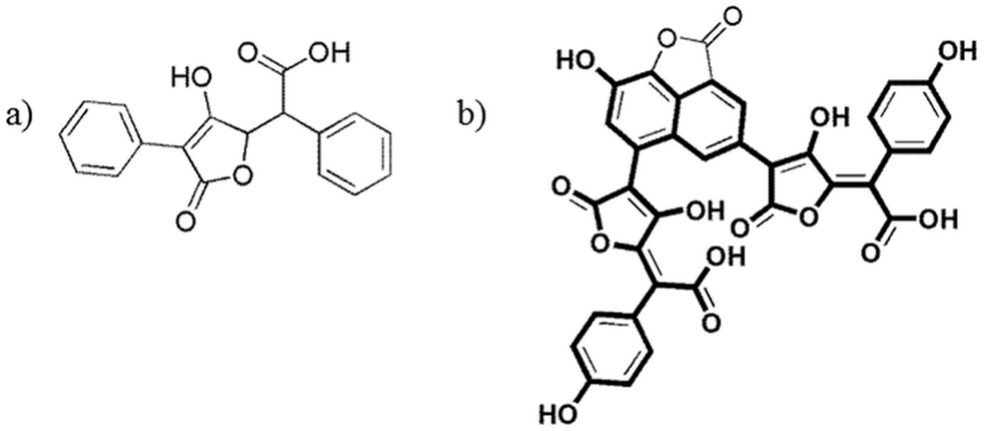
a) The structure of pulvinic acid; b) the structure of norbadione A; two pulvinic acid motives in the structure of norbadione A are bolded.

Le Roux et al. discovered that a pigment norbadione A from the edible mushroom *Xerocomus badius* is a potent natural antioxidant and is especially interesting due to its capacity to reduce the toxicity of ionizing radiation, since the number of known compounds with this ability is rather limited [3]. Unfortunately the compound was found to be cytotoxic, but still presented a promising lead for the development of less toxic derivatives. The pulvinic acid scaffold, which is an important structural motif of norbadione A (Fig 1), was used as a starting point for the synthesis of 18 related compounds, whose experimental evaluation confirmed their antioxidant activity [4]. Another series 28 pulvinic acid derivatives was experimentally evaluated for their antioxidant capacity, and the obtained data was used for the construction of quantitative structure-activity relationship (QSAR) models, which additionally pointed out coumarines as potential antioxidants [5].

In a subsequent study 79 new pulvinic acid derivatives, together with 23 coumarine derivatives, were evaluated for their ability to prevent the degradation of thymidine under three different sources of free radical species (Fenton reaction, UV radiation, gamma radiation). Based on these experimental data, we have derived several QSAR models for the prediction of the antioxidant capacity using different modeling techniques and feature selection approaches [6]. The models were constructed and validated in accordance with the principles set out by The Organisation for Economic Co-operation and Development (OECD) for the validation of QSAR models [7].

**Fig 2:**
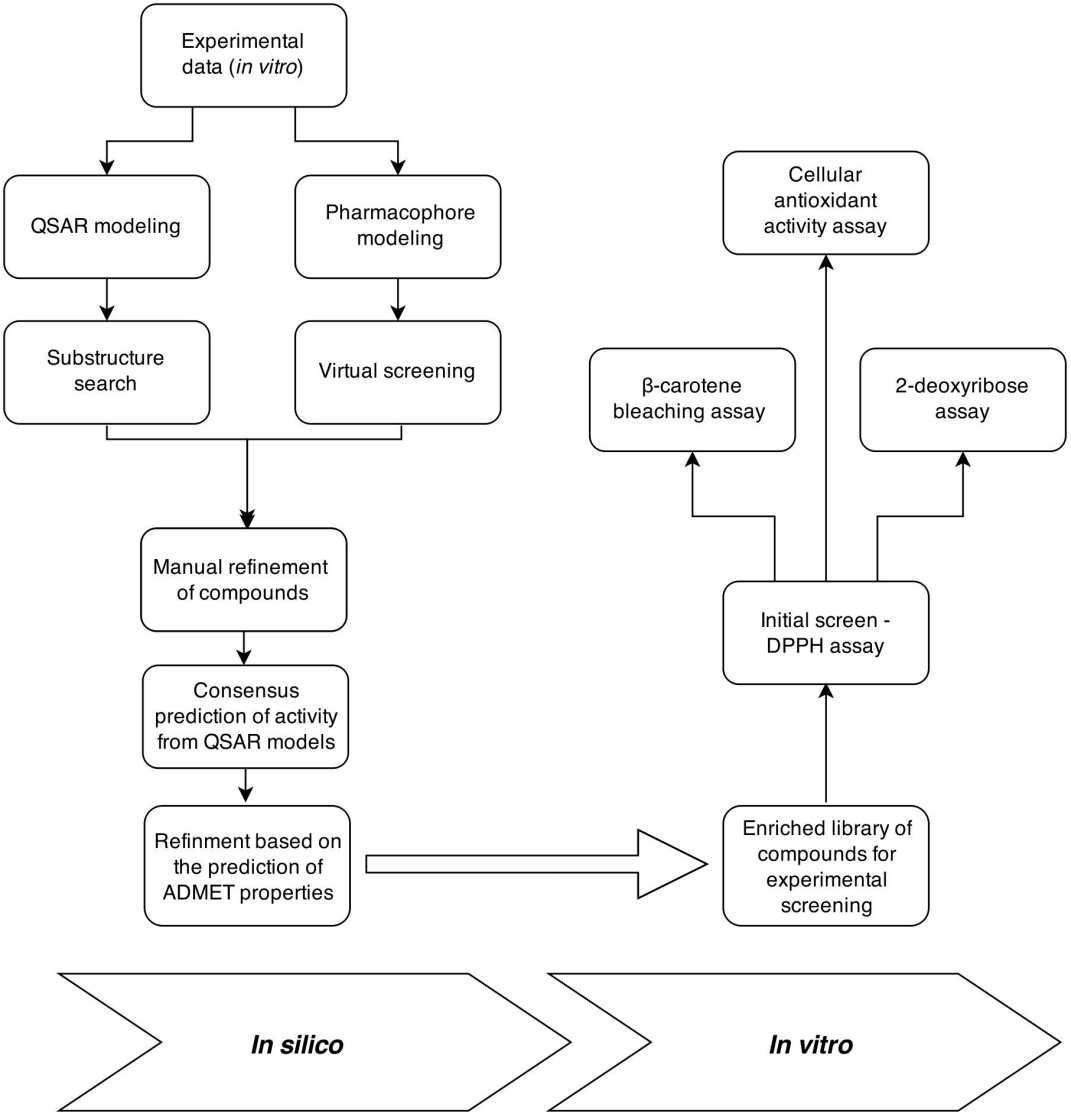
The workflow of the presented study, combining *in silico* and *in vitro* approaches.

In the presented work we utilized the combination of *in silico* and *in vitro* approaches directed towards the discovery of novel potent antioxidants on the basis of pulvinic acid and coumarine derivatives. The workflow of our study is depicted in Fig 2. The *in silico* part began with the virtual screening of commercial compounds libraries, either by utilizing the 2D substructure search or by the use of the ligand-based pharmacophore model derived from our starting antioxidant compounds. Next, the obtained hits were manually refined (a detailed explanation is presented under Results and discussion) and the antioxidant activity of the selected compounds was predicted using the consensus results from our previously developed QSAR models [6]. A couple of pharmacokinetic parameters of the compounds (absorption, solubility) and some toxicity parameters (hepatotoxicity, CYP2D6 inhibition, mutagenicity, carcinogenicity, developmental toxicity) were also evaluated using commercially available models. On the basis of the combined *in silico* results, a final enriched library of compounds for the experimental screening was created. The initial experimental evaluation was carried out using the DPPH assay and the most promising compounds were subsequently investigated by 2-deoxyribose assay, β-carotene bleaching assay and the cellular antioxidant activity assay. In every step of the experimental evaluation the activities of novel compounds were compared to the activities of standard antioxidants. We were successful in our aim of discovering novel potent antioxidants, since almost half of the compounds selected *in silico* displayed *in vitro* antioxidant activity.

## Results and Discussion

### 2.1. *In silico* experiments in search of novel compounds with antioxidant activity

A diverse range of ligand-based *in silico* tools is available that can be used for the design of enriched virtual compound libraries; this usually results in a much greater success rate of a drug design campaign in comparison with the random selection of compounds [8]. We have utilized the substructure search of compound virtual libraries parallel to the virtual screening using a ligand-based pharmacophore model, and finally several QSAR models were employed to predict the antioxidant activity of selected virtual hit compounds.

#### 2.1.1. Virtual screening of libraries of commercially available compounds

In our previous study we have developed several QSAR models for the prediction of the antioxidant activity; three different antioxidant activities have been modeled, corresponding to three different sources of oxidative stress: Fenton reaction, UV radiation and gamma radiation [6].

While QSAR models are a great tool for predicting an unknown activity of a compound, they cannot be always directly applied for large scale virtual screening experiments. Shen et al. for example suggested an elegant procedure for the direct application of QSAR models for screening of compound libraries utilizing the so-called descriptor pharmacophores [9]. While this is a very appealing approach for models using only 2D descriptors, in our case this methodology was not feasible due to the enormous amount of manual input that would be needed, since our QSAR models include descriptors that require a force-field energy minimization prior to their calculation, and in some cases also quantum-chemical descriptors, which require an even more demanding semi-empirical energy minimization. Moreover, after each energy minimization the structures need to be visually inspected to ensure no bonds were broken and no new bonds were formed, which would be practically impossible for entier virtual libraries. Thus, an initial selection of molecules had to be made prior to the use of QSAR models for the prediction of antioxidant activities. Our approach utilized the 2D substructure search using the eMolecules on-line engine [10]. The substructure search was performed with the emphasis on finding hits, which are structurally diverse from pulvinic acid and coumarine derivatives, used for the QSAR models development [6], but still similar enough to these compounds to ensure that at least some of the obtained hits would fall in the applicability domains (AD) of the developed models, which represents the chemical space for which the models predictions can be regarded as reliable [11]. More details on the 2D substructure search are provided in section 4.1.2.

When dealing with the design of compounds that do not have a specific target or the target is unresolved, QSAR models can be complemented by another well-established ligand-based approach, which is the construction of a ligand-based pharmacophore model [12]. The official IUPAC definition defines a pharmacophore as an ensemble of steric and electronic features, which are necessary for the optimal interaction of ligands with a specific biological target structure in order to trigger (or block) its biological response. While we are aware that strictly by definition pharmacophore models can be constructed only for compounds which actually have a specific biological target, in a broader sense the pharmacophore modeling approach can be also utilized to derive generalized three-dimensional spatial arrangements of features, required for a particular activity. In this sense, a pharmacophore model can be seen as the highest common denominator of a series of active compounds [13]. This approach is also in accordance with the IUPAC recommendations for the development of pharmacophore models, which avoid the mentioning of a biological target and state simply that the generation of a pharmacophore is a procedure, where the most important common structural features relevant for a given biological activity are extracted from a series of compounds with a similar mechanism of action. Moreover, several published studies had already applied pharmacophore modeling also to compounds without a specific target, such as antioxidants [14–17], halogenated anesthetics [18] and drugs with non-specific binding to hepatic microsomes [19].

**Fig 3:**
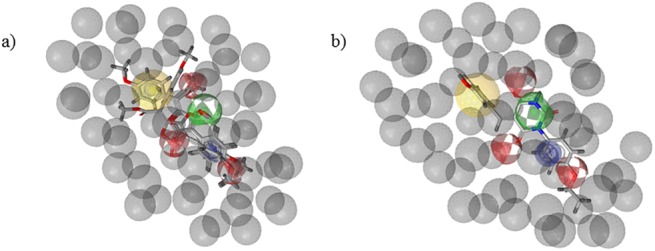
a) The derived pharmacophore model, together with aligned four active pulvinic acid derivatives (IDs: 11, 15, 36, 49 [6]), used in the model development; b) hit compound 13 from the barbituric acid chemical class, aligned to the derived pharmacophore model; this represents a successful scaffold hop from the pulvinic acid scaffold. Yellow sphere–area of hydrophobic interactions; red spheres–hydrogen bond acceptors; green sphere–hydrogen bond donor; blue circle–aromatic ring; grey spheres–exclusion volume spheres.

To further enrich the pool with additional compounds that would be structurally diverse from the chemical class of pulvinic acids, a validated ligand-based pharmacophore model was used in the screening of our in house virtual library, consisting of about two million commercially available compounds from the vendors Vitas-M and ChemDiv. The final pharmacophore model derived on the basis of four selected pulvinic acid derivatives was composed of 6 pharmacophore features: a hydrogen bond donor, three hydrogen bond acceptors, aromatic ring and an area of hydrophobic interactions. The features were surrounded by exclusion volume spheres to limit the space available for the investigated molecules (Fig 3). More details on the development of the pharmacophore model and its application in the virtual screening are provided in section 4.1.3.

The 2D substructure search resulted in 239 novel compounds (provided in the Supporting Information as S1 File), while 271 hits were obtained from the virtual screening (provided in the Supporting Information as S2 File). The hits were merged, resulting in a final pool of 510 compounds for the next *in silico* step in our study.

#### 2.1.2. Prediction of the antioxidant activity using the developed QSAR models

Each compound had its activity predicted with all the selected QSAR models (details on the selection of suitable models are given in section 4.1.1.). Then the consensus of predictions was established in two consecutive steps: first, the predictions of different models for the same activity (Fenton, UV or gamma) were used in consensus, and only the compounds with sufficient average predicted activities proceeded to the next cycle. There the consensus between different antioxidant activities was applied, where only the compounds with sufficiently high predicted activities for at least two of the modeled activities were selected, in order to highlight compounds with a general antioxidant activity and not only the specific ones that the QSAR models were developed to predict. The reasons were two-fold: i) an antioxidant that is active under various oxidizing conditions is generally preferred to one that is active only under specific conditions; ii) due to the unavailability of specific equipment the original assays of the protection of thymidine [20,21] could not be replicated for the novel compounds, so an alternative was to predict a general antioxidant activity and then evaluate it with some other well-established antioxidant assays.

**Fig 4:**
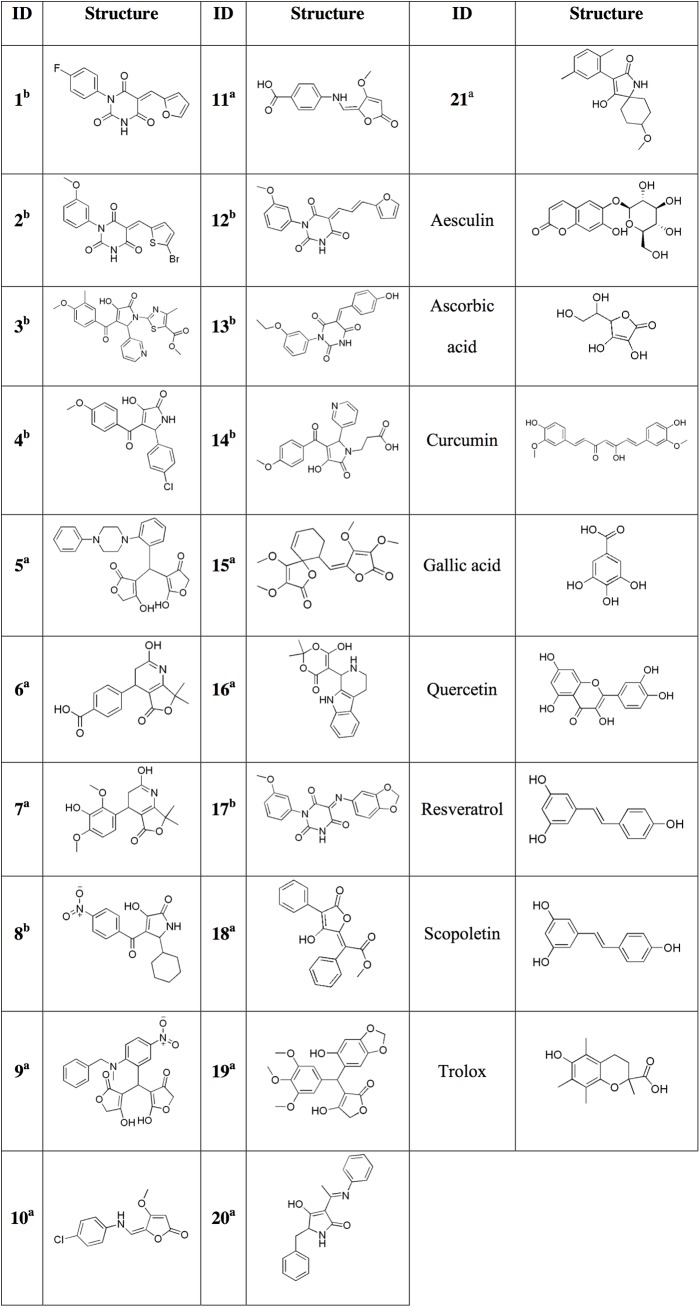
Structures of the novel compounds (1–21) and standard antioxidants, purchased for experimental testing. ^a^ Compounds selected by the substructure search; ^b^ Compounds selected by pharmacophore screening.

Next, a few external models provided in the software BIOVIA Pipeline Pilot^®^ version 9.2 (San Diego, CA) were applied to the proposed compounds for the prediction of their aqueous solubility, hepatotoxicity, plasma protein binding, metabolic enzyme CYP2D6 binding, mutagenicity, and ability to cross the blood–brain barrier. On the basis of all the predictions and by following the general guidelines for drug-likeness, a prioritization list of the most promising compounds for experimental testing was created. 21 novel compounds (Fig 4) were obtained from commercial sources; 12 of these were found by a substructure search, while the remaining 9 were selected by pharmacophore screening; the pharmacophore model enabled us to make a scaffold hop from pulvinic acid derivatives by including derivatives of barbituric acid and 3-hydroxy-1,5-dihydro-pyroll-2-one. 8 standard antioxidants were also purchased for the sake of comparison with the novel compounds and validation of the experimental procedures.

### 2.2. *In vitro* experimental evaluation of the antioxidant activity

Antioxidants are sometimes capable of acting through different mechanisms, depending on the reaction system, or even by multiple mechanisms in a single system. Moreover, different radical sources may cause different responses from a single antioxidant. This is the reason why no single assay is able to accurately evaluate the true antioxidant capacity, and consequently several different assays are usually employed to better assess the capacity of novel antioxidants [22,23].

We found that 10 of the 21 novel compounds exhibited antioxidant capacity in at least one *in vitro* test that is comparable or even better than the activities of some standard antioxidants. Compounds **5**, **7**, **9**, **11** and **12** were found to be active in all four *in vitro* assays, compound **10** was active in three assays, compounds **13**, **18** and **21** were active in two assays, while compound **19** was active only in one assay. However, it should be noted that due to the small purchased quantity, compounds **18**, **19** and **21** could not be evaluated with all the proposed assays. Moreover, none of the standard antioxidants displayed antioxidant activity in all four assays, so we can conclude that some of the novel compounds even surpass the standard antioxidants in this respect.

#### 2.2.1. DPPH assay

The 2,2-diphenyl-1-picrylhydrazyl (DPPH) assay is one of the most popular assays for the evaluation of the antioxidant capacity of compounds due to its simplicity, low cost and relative speed [24]. The basis of the assay is that DPPH, which is a stable nitrogen centered radical with an intensive violet color in a solution (and a characteristic absorption maximum at 516 nm), is reduced to 1,1-diphenyl-picryl hydrazine (DPPH_2_) by transferring a hydrogen from the compound being tested, which results in a reduced absorbance at 516 nm [25].

A possible drawback of the DPPH assay is the fact that the reaction is reversible to some extent, so the measured antioxidant capacity may be lower than it should be [26]. Another possible problem is the steric accessibility, since larger molecules may not be able to access the DPPH radical site [27].

First, the standard antioxidants were tested for the purpose of comparison with novel compounds, and also to indicate the quality of our measurements by comparing the results to the literature data. According to Table 1, the calculated EC_50_ values from our experiment match quite well with the published data, providing us with additional confidence in our experimental results.

**Table 1:** The calculated EC_50_ values (95% confidence interval) of novel compounds and standard antioxidants in the DPPH assay, together with the literature data on the activity of standard antioxidants. The EC_50_ curves are provided in the Supporting Information in the S1 Table.

Novel compound	EC_50_ exp. [μM]	Standard	EC_50_ exp. [μM]	EC_50_ lit. [μM]
**5**	11.0–14.0	Ascorbic acid	21.0–23.0	28.0 [28], 10.0 [26], 15.0 [29], 33.0 [30]
**7**	18.0–21.0	Gallic acid	5.0–6.0	5.1 [31], 4.2 [26]
**9**	18.0–22.0	Quercetin	4.0–5.0	3.3 [32], 4.8 [29], 4.3 [33]
**10**	18.0–23.0	Scopoletin	110.0–140.0	180.0 [34], 190.0 [35]
**11**	16.0–23.0	Aesculin	(1.1–3.4)**×**10^3^	2.4**×**10^3^ [36], 3.7**×**10^3^ [37]
**12**	19.0–25.0	Trolox	14.0–18.0	19.0 [38], 18.0 [39]
**13**	15.0–16.0	Curcumin	12.0–14.0	39.0 [40], 32.0 [30]
**18**	23.0–27.0	Resveratrol	13.0–18.0	35.0 [41],38.0 [42]
**19**	27.0–35.0			
**21**	20.0–23.0			

10 of the 21 purchased novel compounds were found to be active in the DPPH assay. Since this was used as a mean of an initial experimental screening, only these compounds proceeded to be tested with the other assays. All the active compounds obey the Lipinski rules [43], and with the exception of compound **9** also the Veber rules [44], indicating that these compounds have a high probability of being well absorbed after oral intake. The calculated Log D_7.4_ values for the majority of active compounds (with the exception of compound **11**) are in the range between 1 and 3, which is ideal for drug candidates, since these compounds should generally possess a good balance of solubility and passive diffusion permeability, resulting in a good intestinal absorption; moreover, the metabolism should be generally minimized, due to lower binding to metabolic enzymes [45].

**Fig 5:**
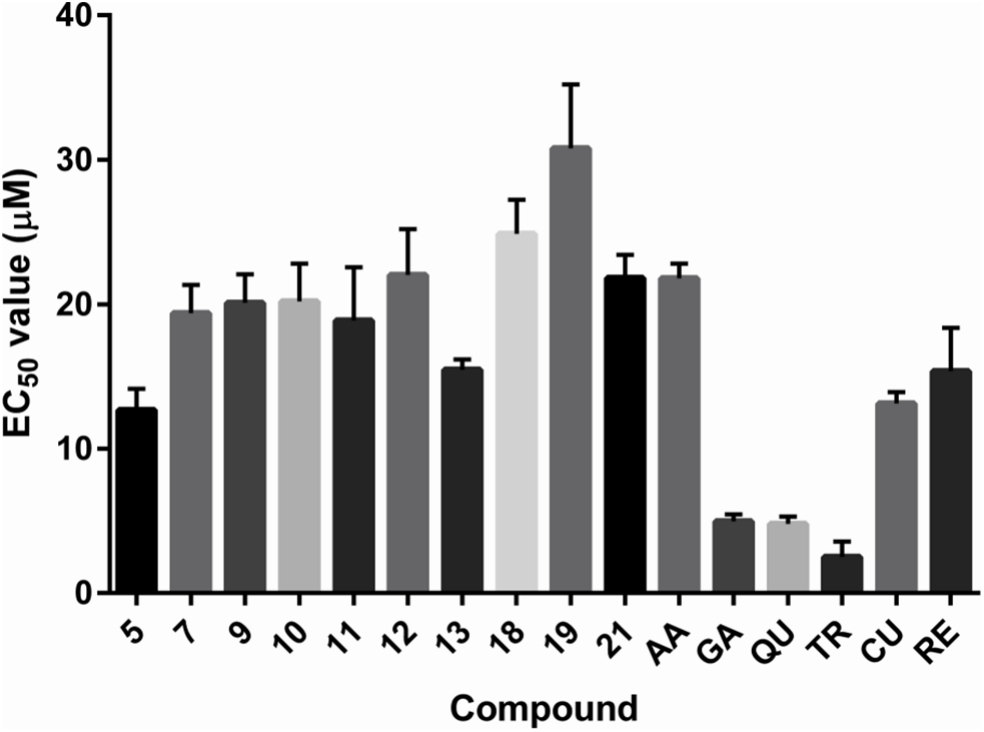
Histogram of the experimentally determined EC_50_ values in the DPPH assay. AA–ascorbic acid; GA–gallic acid; QU–quercetin; TR–Trolox; CU–curcumin; RE–resveratrol. The EC_50_ values of scopoletin and aesculin are not depicted, due to being much larger.

The EC_50_ values of the novel compounds do not differ much (all are in the range of 10–35 μM), and are comparable to the EC_50_ values of ascorbic acid, Trolox, curcumin and resveratrol, and much higher than the EC_50_ values of aesculin and scopoletin (Fig 5). However, it should be noted that only compound **19** was able to reach the steady state within one hour, while all other novel compounds required a 24 hour incubation period for the reaction to fully take place. On the other hand, several standard antioxidants (aesculin, scopoletin, curcumin and resveratrol) also required 24 hours of incubation to reach the steady state of the reduced DPPH.

**Fig 6:**
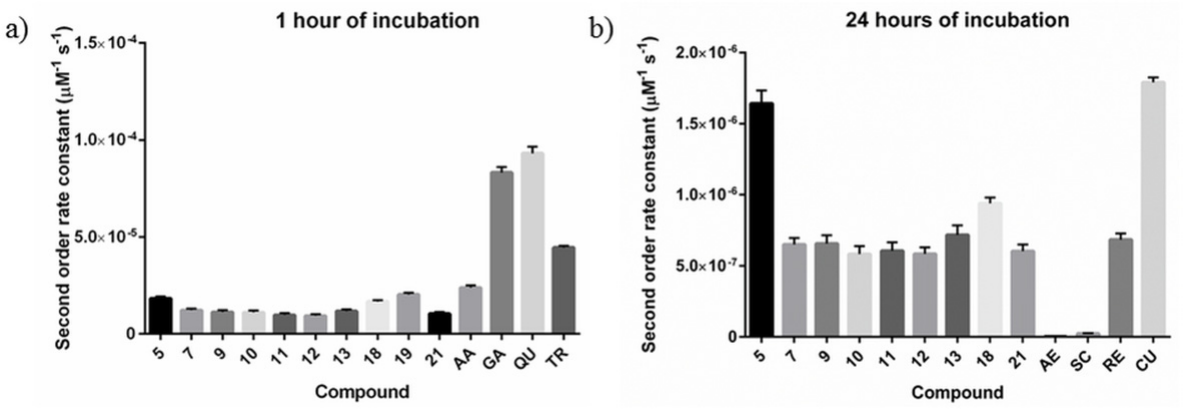
a) Histogram of the second-order rate constants determined after 60 minutes from the initiation of the reaction; b) histogram of the second-order rate constants determined after 24 hours from the initiation of the reaction. AA–ascorbic acid; GA–gallic acid; QU–quercetin; TR–Trolox; AE–aesculin; SC–scopoletin; RE–resveratrol; CU–curcumin.

The kinetic behavior of the compounds was evaluated by calculating the second-order rate constants for the reaction with DPPH during the first 60 minutes, and also during the total 24 hours of incubation, where appropriate. When comparing the rate constants of the novel compounds during the first 60 minutes to the standard fast antioxidants (such as ascorbic acid, gallic acid, quercetin and Trolox), we found the kinetic behavior of the novel compounds to be slower, but still within the same order of magnitude. In the case of the rate constants during the total 24 hours, we found the kinetic behavior of the novel compounds to be similar to that of resveratrol and curcumin, and much more rapid than the action of aesculin and scopoletin (Table 2 and Fig 6).

**Table 2:** The calculated second-order constant rates of novel compounds and standard antioxidants, determined after 60 minutes or after 24 hours from the initiation of the reaction.

Compound	Constant rate exp. [10^−5^ μM^-1^ s^-1^] (60 minutes)	Constant rate exp. [10^−7^ μM^-1^ s^-1^] (24 hours)
**5**	1.8 ± 0.1	16.4 ± 0.9
**7**	1.2 ± 0.1	6.5 ± 0.5
**9**	1.1 ± 0.1	6.6 ± 0.6
**10**	1.1 ± 0.1	5.8 ± 0.6
**11**	1.0 ± 0,1	6.1 ± 0.6
**12**	0.9 ± 0.1	5.8 ± 0.5
**13**	1.2 ± 0.1	7.2 ± 0.7
**18**	1.7 ± 0.1	9.4 ± 0.4
**19**	2.0 ± 0.1	/
**21**	1.1 ± 0.1	6.0 ± 0.5
Ascorbic acid	2.4 ± 0.1	/
Gallic acid	8.3 ± 0.3	/
Quercetin	9.3 ± 0.3	/
Trolox	4.5 ± 0.1	/
Aesculin	/	(20.0 ± 5.0)**×**10^−3^
Scopoletin	/	(2.0 ± 0.3)**×**10^−1^
Resveratrol	/	6.9 ± 0.4
Curcumin	/	17.9 ± 0.4

#### 2.2.2. 2-deoxyribose assay

The 2-deoxyribose (2-DR) assay determines the ability of an antioxidant to protect 2-deoxyribose from degradation by scavenging *in situ* formed hydroxyl radicals. Although the method is simple and inexpensive, the results are comparable with electron paramagnetic resonance (EPR) spectroscopy [46]. When 2-DR is degraded malondialdehyde-like products are formed, which produce a pink chromogen after heating in the presence of thiobarbituric acid (TBA). These degradation products are in general referred to as thiobarbituric acid reactive substances (TBARS), and their formation can be assessed spectrophotometrically [47].

The total content of TBARS in the 2-DR assay is composed of TBARS from specific 2-DR degradation caused by the hydroxyl radical, TBARS produced from heat induced 2-DR degradation during the boiling phase of the experiment, and possible TBARS from the degradation of the evaluated compound itself. Since we are interested only in the specific 2-DR degradation by the hydroxyl radical, appropriate blind samples are needed to evaluate other TBARS. We came across several different suggestions for the blind sample [46–49], but found none of these appropriate. Thus a novel blind sample was designed: first, the nonspecific 2-DR degradation (induced by the boiling phase) was quantified by subtracting the absorbance of the blind sample BL1 (without 2-DR, without the antioxidant, without Fe^3+^, without H_2_O_2_) from the absorbance of the blind sample BL2 (without the antioxidant, without Fe^3+^, without H_2_O_2_). Furthermore, since we found some of our compounds were also degraded to some extent to produce TBARS, this was quantified by another blind sample BL3, containing all the reagents and the tested compound, without the addition of 2-DR; all TBARS from BL3 can be ascribed to the degradation of the tested compound. Finally, the absorbance of TBARS arising from the specific 2-DR degradation from the hydroxyl radical attack was calculated by the following formula:

$$A_{sample}=A_{measured}-A_{BL3}-(A_{BL2}-A_{BL1})$$ (1)

where A_sample_ stands for the treated absorbance of the sample (that should be used in further calculations), A_measured_ is the directly measured absorbance of each sample, and A_BL1_, A_BL2_ and A_BL3_ represent the absorbances of blind samples BL1, BL2 and BL3, respectively.

**Fig 7:**
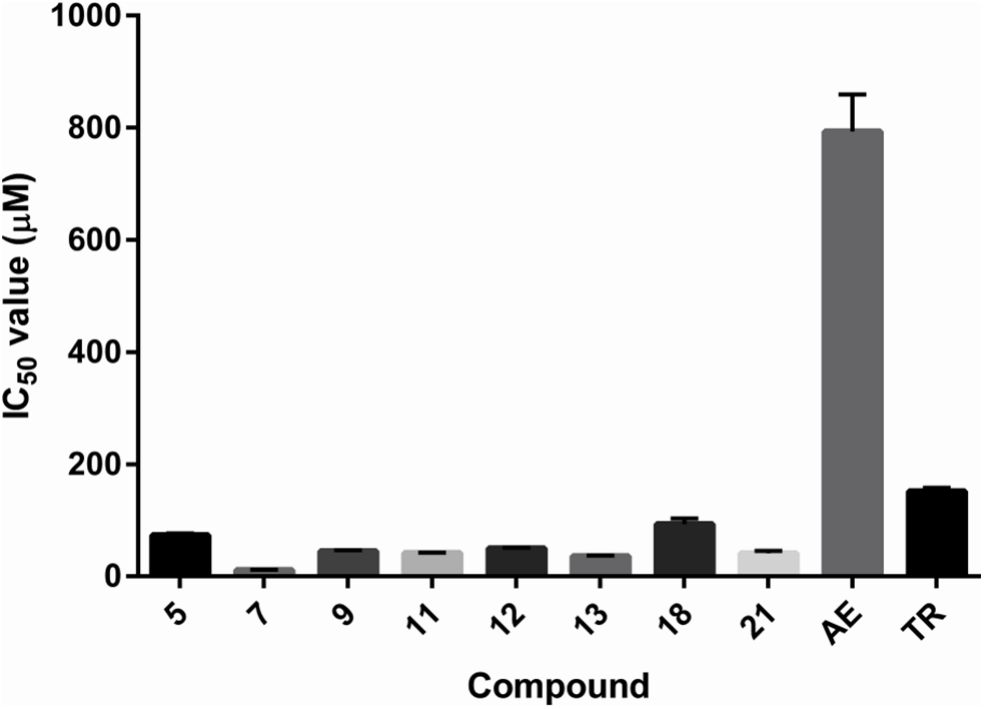
Histogram of the experimentally determined IC_50_ values in the 2-DR assay. TR–Trolox; AE–aesculin.

Most of the compounds that were active in the DPPH assay were also displayed activity in the 2-DR assay, with the exception of compound **10**, that could not be evaluated due to its low water solubility, and compound **19**, which was found to have a pro-oxidant effect in the 2-DR assay system. The reason could be that the compound **19** is able to recycle Fe^3+^ back to Fe^2+^, thus promoting the Fenton reaction [50]. The pro-oxidant effect was also observed with some of the standard antioxidants, more precisely gallic acid, resveratrol, curcumin and quercetin. The spectrum of activities was a bit wider than in the case of the DPPH assay, with the IC_50_ values ranging from approximately 10 to 100 μM (Table 3 and Fig 7). Novel compounds clearly outperformed the standard antioxidants Trolox and aesculin regarding the antioxidant activity. The determined IC_50_ values of aesculin and Trolox correspond well with the published literature data, giving us confidence in the accuracy of our measurements.

**Table 3:** The calculated IC_50_ values (95% confidence interval) of novel compounds and standard antioxidants in the 2-DR assay, together with the literature data on the activity of standard antioxidants. The IC_50_ curves are provided in the Supporting Information in the S1 Table.

Compound	IC_50_ exp. [μM]	Standard	IC_50_ exp. [μM]	IC_50_ lit. [μM]
**5**	70.0–77.0	Trolox	150.0–160.0	280.0 [51], 130.0 [52], 520.0 [53]
**7**	10.0–12.0	Aesculin	730.0–860.0	330.0 [54]
**9**	43.0–47.0			
**11**	40.0–43.0			
**12**	48.0–51.0			
**13**	35.0–37.0			
**18**	84.0–103.0			
**21**	37.0–46.0			

To better evaluate the antioxidant properties in the 2-DR assay, we attempted to calculate the second-order rate constants for these reactions [47]. The prerequisite is that the plot of 1/A versus the compound concentration is linear, and we found that for the majority of the tested compounds (except aesculin) these plots were non-linear. The calculated second-order rate constant for aesculin was (9,05 ± 0,48)**×**10^9^ M^-1^ s^-1^. The non-linear competition plots for other compounds suggest they are able to react directly with hydrogen peroxide, thus diminishing the rate of hydroxyl radicals generation [47]. On the other hand, this indicates these compounds actually possess a dual antioxidant mode of action: they are not only effective scavengers of hydroxyl radicals, but also scavengers of hydrogen peroxide. To confirm this, a quick test of hydrogen peroxide scavenging capacity was carried out as described by [55], with small modifications. All the evaluated novel compounds (**5**, **7**, **9**, **11**, **12**, **13**) were found to actually possess low to moderate hydrogen peroxide scavenging capacity, so we expect the same to be true also for other compounds with nonlinear competition plots (**18**, **21**, and Trolox).

#### 2.2.3. β-carotene bleaching (BCB) assay

The β-carotene bleaching assay is also a commonly used method for assessing the antioxidant capacity. The assay is based on the discoloration of β-carotene, caused by an attack of lipid peroxide radicals (LOO^•^), generated by the oxidation of linoleic acid; the addition of an effective antioxidant reduces the discoloration, which can be quantified spectrophotometrically. In the original assay the oxidation is induced thermally (at 50°C) [56], but the method is time consuming, hard to reproduce, and quite non-specific due to the involvement of thermal degradation of β-carotene. Thus another variation of this assay was suggested, utilizing the enzyme LOX to induce the fatty acid oxidation; this method is much faster, it does not involve non-specific β-carotene degradation, and it takes place at room temperature [57]. This new method was employed also in this study, with some slight modifications.

The BCB assay system is based on the oxidation of lipid micelles in an aqueous environment, thus offering a better representation of the true biological systems in comparison with the DPPH and 2-DR assays [56]. The possible shortcomings are the assay takes place in emulsion conditions, so the activity of hydrophilic antioxidants may be reduced due to their moving to the water phase, while lipophilic antioxidants may show greater antioxidant activity [58].

**Fig 8:**
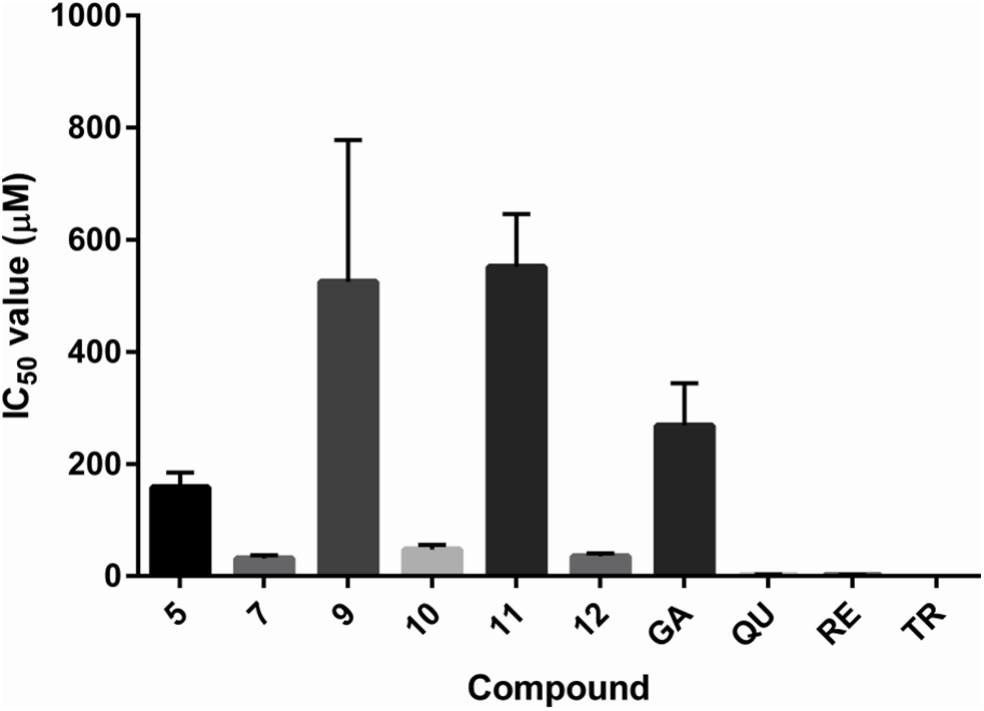
Histogram of the experimentally determined IC_50_ values in the BCB assay. GA–gallic acid; QU–quercetin; RE–resveratrol; TR–Trolox.

Active compounds from the DPPH assay mostly displayed activity also in the BCB assay; the exceptions were compounds **13** and **18** with no effect, while ascorbic acid displayed a slight pro-oxidant activity. Active novel compounds have a relatively wide range of IC_50_ values, spanning from 30 to 780 μM (Table 4 and Fig 8). Their activities are comparable to (or even slightly better) than that of gallic acid, while quercetin, resveratrol and Trolox proved to be much more potent LOO^•^ scavengers.

**Table 4:** The calculated IC_50_ values (95% confidence interval) of novel compounds and standard antioxidants in the BCB assay, together with the literature data on the activity of standard antioxidants. The IC_50_ curves are provided in the Supporting Information in the S1 Table.

Compound	IC_50_ exp. [μM]	Standard	IC_50_ exp. [μM]	IC_50_ lit. [μM]
**5**	140.0–190.0	Gallic acid	210.0–340.0	62.0 [32], 2.6×10^3^ [59]
**7**	270.0–370.0	Quercetin	2.2–2.6	5.4 [60], 1.8 [61]
**9**	350.0–780.0	Resveratrol	1.7–2.0	No data found
**10**	40.0–56.0	Trolox	(3.7–4.3)**×**10^−1^	0.6 [62]
**11**	470.0–650.0			
**12**	31.0–41.0			
**13**	No effect			
**18**	No effect			

**Fig 9:**
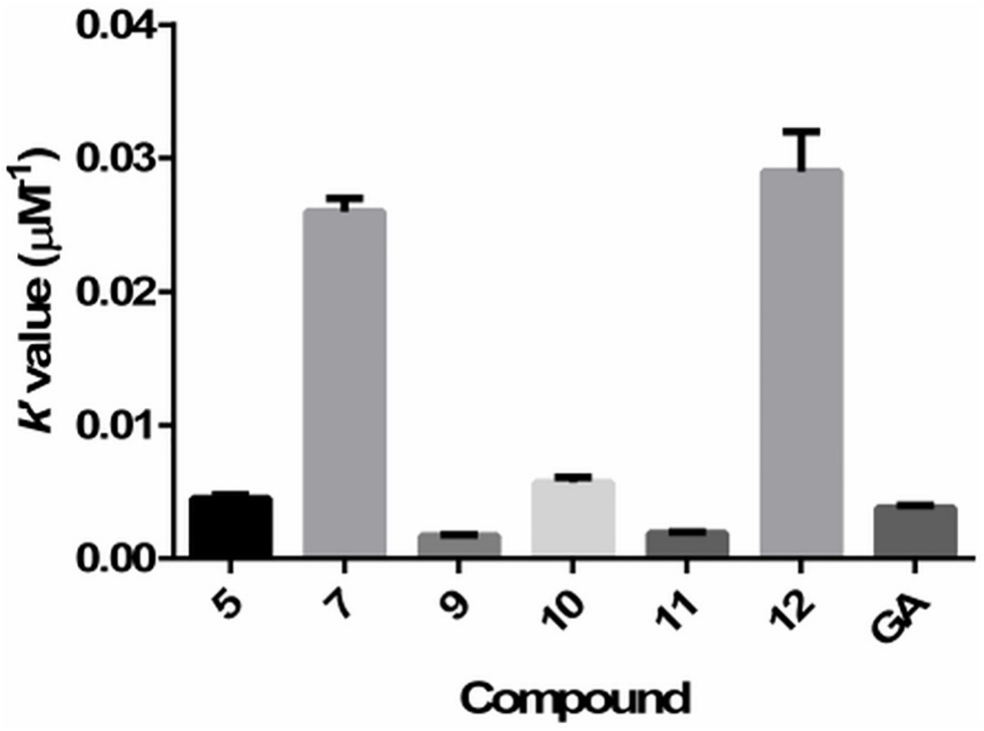
Histogram of the calculated *k’* values in the BCB assay. Rate constants of quercetin, resveratrol and Trolox are not depicted due to being much higher. GA–gallic acid.

An attempt was made to evaluate also the kinetics of the reactions of LOO^•^ with the tested compounds. Since the principles behind BCB assay and the 2-DR assay are quite similar (in both cases, an *in situ* source of radicals attacks the probe, that should be protected by the evaluated compound, we decided to use the same procedure as for the calculation of rate constants in the 2-DR assay [47]. By plotting the values of 1/∆A (where ∆A represents the difference between the initial and the final absorbance) versus the antioxidant concentration, linear plots were obtained, and the rate constants can be obtained from the slopes by the following equation:

$$k=slope\times k_{\beta C}\times [\beta C]\times A^{0}$$ (2)

where *k*_βC_ represents the rate constant of the reaction of β-carotene with LOO^•^, [βC] is the concentration of β-carotene and A^0^ stands for the control absorbance. However, since we found no literature data on value of *k*_βC_, the exact rate constants could not be calculated. However, given that the values of *k*_βC_ and [βC] are constant, the kinetic behavior of the tested compounds can still be compared just from the product of the slope and A^0^, which we named *k’*. The calculated *k’* values are presented in Table 5 and graphically depicted in Fig 9.

**Table 5:** The calculated *k’* values of novel compounds and standard antioxidants in the BCB assay.

Compound	*k’* [μM^-1^]
**5**	(4.5 ± 0.3)×10^−3^
**7**	(2.6 ± 0.1)×10^−2^
**9**	(1.7 ± 0.1)×10^−3^
**10**	(5.7 ± 0.4)×10^−3^
**11**	(1.9 ± 0.1)×10^−3^
**12**	(2.9 ± 0.3)×10^−2^
Gallic acid	(3.8 ± 0.2)×10^−3^
Quercetin	(3.5 ± 0.1)×10^−1^
Resveratrol	(3.7 ± 0.2)×10^−1^
Trolox	2.1 ± 0.1

Based on the calculated *k’* values, the kinetic behavior of active novel compounds in the BCB assay is comparable to that of gallic acid, with the exception of compounds **7** and **12**, whose reaction rates are almost 10-times faster. On the other hand, other standard antioxidants (quercetin, resveratrol and Trolox) proved to be much faster acting antioxidants (100–1000 times faster). It is interesting to note that while compound **12** seems to be the most active novel compound, it also has the slowest kinetic behavior.

#### 2.2.4. Cellular antioxidant activity (CAA) assay

There are many shortcomings to various *in vitro* assays, especially not accounting for the bioavailability and metabolism of compounds, as well as performing them at non-physiological pH and temperatures. On the other hand, animal models and human studies are too expensive and time consuming to be routinely used for preliminary screening of antioxidants. The cellular antioxidant activity (CAA) assay employs a biologically relevant model system with physiological conditions, which does not consider only the antioxidant activity, but also the cellular uptake, metabolism and distribution. Thus it represents a suitable compromise between classical *in vitro* and *in vivo* experiments; while it mirrors the complex biological systems much better than test tube methods, it is still fast and cheap enough to be used as a screening tool [63,64].

The assay is based on the fact that when living cells are exposed to DCFH-DA, it diffuses through the cellular membrane and is then transformed by intracellular esterases to a polar 2′,7′-dichlorodihydrofluorescin (DCFH), which is unable to escape the cells. An inductor of oxidative stress is then introduced; while the produced free radicals attack the membrane and start a chain propagation reaction, they also oxidize the intracellular DCFH to 2′,7′-dichlorofluorescin (DCF), which can be quantified due to its fluorescence. The addition of an antioxidant into the system should prevent/lessen the oxidation of DCFH, thus reducing the measured fluorescence [63].

Each of the compounds was evaluated at its highest non-toxic concentration and half of that concentration. The activities at a given concentration were expressed with a CAA value, which can range from 0 to 100, where the CAA value of 0 represents no antioxidant activity, while the CAA value of 100 represents the complete protection from the attack of oxidizing species. On the other hand, the CAA values can also take negative values, which indicate the pro-oxidant activity of such compounds. Table 6 represents the calculated CAA values for the evaluated novel compounds and standard antioxidants ascorbic acid and gallic acid.

**Table 6:** The calculated CAA values of novel compounds and the standard antioxidants in the CAA assay.

Compound	CAA value (62.5 μM)	CAA value (31.25 μM)	CAA value (15.625 μM)
Ascorbic acid	71.0	79.9	/
Gallic acid	76.9	85.1	/
**5**	-26.9	62.8	/
**7**	41.5	68.5	/
**9**	73.5	79.8	/
**10**	65.0	76.9	/
**11**	75.4	47.6	/
**12**	/	-152.3	-258.5
**13**	77.1	46.0	/

Most evaluated compounds displayed some degree of antioxidant activity, with the exception of compound **12**, which was a pro-oxidant at both tested concentrations, and compound **5**, which was an antioxidant when used at 31.25 μM, but turned pro-oxidant when the concentration was raised to 62.5 μM. Compounds **9** and **10** displayed the greatest activities, comparable to those of ascorbic acid and gallic acid.

An interesting notion is that with the exception of compounds **11**, **12** and **13**, all the compounds were more active when used at a lower concentration; this indicates that when higher concentrations are used, the character of the compounds starts partially turning from antioxidant to pro-oxidant, resulting in a reduced protection from free radicals. In the case of compound **5**, when the concentration was raised from 31.25 μM to 62.5 μM, the pro-oxidant activity probably already prevailed over the antioxidant, thus resulting in a negative CAA value. Compound **12** was found to be a pro-oxidant at both tested concentrations; the curious phenomenon was that the pro-oxidant activity was more pronounced when the compound was used at a lower concentration, and not vice versa, which was the case with the majority of other compounds.

In the traditional CAA assay, the production of ROS is induced by 2,2’-azobis (2-amidinopropane) dihydrochloride (ABAP), that spontaneously decomposes to peroxyl radicals, which attack the cell membrane and start the chain propagation reaction. ABAP decomposition can occur on the cell surface or inside the cell, which means that the evaluated antioxidant can interfere with the produced ROS either on the cellular surface or inside the cell. This is why the traditional CAA assay has two variations; it can be performed with or without the buffer wash between the treatments of cells with the evaluated antioxidant and induction of the oxidative stress. If the buffer wash is applied, the antioxidants from the cell surface are removed, and the remaining antioxidant activity can be attributed just to the intracellular fraction of antioxidant.

However, in our case phorbol 12-myristate 13-acetate (PMA) was used instead of ABAP to induce oxidative stress; it is a protein kinase C activator, which in turn activates two important cellular oxidative enzymes, NADPH oxidase [65,66] and xanthine oxidase [67]. This increases the production of ROS in the cell and results in an increased rate of DCFH oxidation in lymphocytes and monocytes [68]. Since PMA induces oxidative stress through the activation of intracellular enzymes, the production of ROS is also predominantly intracellular, and the measured antioxidant activity can be mostly attributed to the intracellular fraction of the antioxidant, even without applying the buffer wash. If a compound is found to be active, this is not only the display of its antioxidant potential, but also an indirect proof of its ability to penetrate into the cells. All the evaluated compounds displayed some kind of activity in the CAA assay (be it anti- or pro-oxidant), so from the bioavailability point of view we can conclude they were all able to cross the cell membrane. Moreover, the displayed intracellular activity also indicates the appropriate distribution of the compounds inside the cells and their relative resistance to the intracellular metabolism.

### 2.3. Interpretation of results and the highlight of most promising novel compounds

Compounds **10** and **11** are structurally very similar; the only difference is that compound **10** possesses a chlorine atom instead of a carboxylic group. This explains the low water solubility of compound **10**, which prevented us from evaluating it with the 2-DR assay, and is probably also the reason for the 10-fold greater activity of compound **10** (log P = 3.5) in comparison with compound **11** (log P = 2.7) in the BCB assay, since lipophilic antioxidants may show greater antioxidant activity due to emulsion conditions in the BCB assay. We assume that the carrier of the antioxidant activity of these compounds is the exocyclic double bond on the position 5 of tetronic acid moiety, or the adjacent secondary amine. Interestingly, while compound **17** possesses this same fragment, it displayed no activity.

Compounds **12** and **13** are both barbituric acid derivatives. We assumed that their antioxidant activity originates from the exocyclic double bond, attached to the barbituric acid scaffold; however, while other barbituric acid derivatives **1**, **2** and **17** possess the same exocyclic double bond as compounds **12** and **13**, none were found to be active. The activity of compound **12** in comparison with compounds **1**, **2** and **17** could be explained by a larger system of conjugated double bonds, which can better stabilize the radical, which is formed after the subtraction of the labile hydrogen from the exocyclic double bond, while the activity of compound **13** could be due to the free phenolic hydroxyl group.

Compounds **6** and **7** share very similar structural features; the main difference is that compound **6** has a carboxylic group on the para position of the aromatic ring, while compound **7** has a hydroxyl group in the same place. This substituent seems to be essential for the antioxidant activity, since compound **6** was found to be inactive, while compound **7** was active.

Compounds **5** and **9** share a high degree of similarity, both possessing two adjacent tetronic acid moieties, which results in their high hydrophilicity with logP values of -0.083 and -0.265, respectively. This is probably the reason for their relatively low activity in the BCB assay.

Compounds **20** and **21** were the only tetronic acid derivatives with the cyclic oxygen atom replaced with nitrogen, while compounds **3**, **4**, **8** and **14** are closely related derivatives of 3-hydroxy-1,5-dihydro-pyroll-2-one. All compounds have an unsubstituted hydroxyl group in the vinylogous acid fragment, which is the presumed source of antioxidant activity; interestingly only compound **21** proved to be active. While in the case of **3**, **4**, **8** and **14** the vinylogous acid fragment is not part of the ring structure and might offer an explanation for the lack of activity, it is intriguing to observe the lack of activity of **20**. Compound **16** is a Meldrum’s acid derivative in the enolic tautomeric form, which shares the vinylogous acid moiety with tetronic acid. We expected this compound also to be active due to the free hydroxyl group in the vinylogous acid fragment, but no activity was observed.

Based on the combined results of the DPPH, 2-DR and BCB assays, compounds that were active in all these assays are **5**, **7**, **9**, **11** and **12**, with the measured activities in the micromolar range. Compounds **5**, **9** and **11** have about 10-fold weaker activity in the BCB assay than the other two assays, but this is probably due to their high hidrophilicity, which reduces the apparent activity in the BCB assay. Compound **10** displayed good activity in DPPH and BCB assays, but its further use could be problematic due to its low water solubility, which was shown in the 2-DR assay.

The results of the CAA assay point that compound **12** can be discarded due to its pro-oxidant cellular activity. Compound **11** displayed good activity at 62.5 μM, but since this is also its highest non-cytotoxic concentration, the potential therapeutic window of this compound would be too narrow. On the other hand, the activities of compounds **5**, **7** and **9** were higher when used at 31.25 μM than 62.5 μM (Table 6). This indicates that at higher concentration, these compounds become partially pro-oxidants, so we might speculate that their activity at lower concentrations would be even better. Thus, we would like to highlight compounds **5**, **7** and **9** as the most promising candidates for further studies.

## Conclusions

In the presented study we successfully identified several promising novel antioxidant compounds using a combination of *in silico* and experimental procedures. In the *in silico* stage, starting from the available experimental data for a relatively small set of antioxidants from the pulvinic acid and coumarine classes, QSAR and antioxidant-based pharmacophore models were constructed and used in the virtual screening of large compound libraries in search for novel compounds with antioxidant activity. After several subsequent refinement steps a small set of 21 novel compounds alongside 8 establish antioxidant compounds was selected for the experimental evaluation.

In the experimental stage the antioxidant activity of the selected compounds was investigated through several well-established *in vitro* assays. The initial DPPH assay identified 10 promising compounds containing tetronic acid and barbiturate scaffolds that were further investigated by 2-deoxyribose assay, β-carotene bleaching assay and the cellular antioxidant activity assay. We established in our assays that the hit compounds **5**, **7** and **9** are effective scavengers of a wide array of free radical species, which shows we were successful in the search for compounds with a general antioxidant activity. Moreover, the results of the cellular antioxidant activity assay indicate that the novel compounds **5**, **7**, **9**, **10**, **11**, and **13** do not just possess the desirable antioxidant activity, but are also able to cross the cellular membrane and withstand the cellular metabolism, which are both important aspects in the design of new drugs.

The derived *in silico* models proved their efficiency by providing an 48% hit-rate of the experimental screening campaign (with 10 of the 21 compounds being active). When we retrospectively compared the number of active hits, obtained from the substructure search of the virtual libraries and pharmacophore screening, the substructure search (8 actives from 12 selected compounds) outperformed the pharmacophore screening (2 actives from 9 selected compounds). However, by using just the substructure search on the basis of similarity with the compounds from our QSAR models, we would have remained confined within the pulvinic acid derivatives chemical space, while the pharmacophore model was successful in predicting the antioxidant activity of some compounds with a barbiturate scaffold, which also displayed promising antioxidant activities.

Since the cellular antioxidant activity assay was not performed using several different concentrations of the evaluated compounds, the IC_50_ values could not be calculated. Furthermore, nothing is known regarding the exact antioxidant mechanism of the presented novel compounds. These are some of the challenges of our future work, which could (together with the presented study) offer new information for paving the way towards novel useful synthetic antioxidants.

We would like to also stress that an innovative methodology for discovering new potent compounds was developed. Indeed, no new methods were discovered, but known methods were combined into a consecutive set of steps that resulted in a selection of reasonably active compounds in the most economical way, saving time and money in comparison with the conventional approaches based on commonly used procedures.

## Materials and Methods

### 4.1. *In silico* methods

#### 4.1.1. QSAR modeling

The QSAR models that were employed for the prediction of antioxidant activity were constructed in a previous study, on the basis of a dataset of 79 pulvinic acid derivatives, 23 coumarine derivatives and 9 structurally non-related compounds, together with the corresponding experimentally determined antioxidant activities from the thymidine protection assay under three different sources of free radical species (Fenton reaction, UV radiation, gamma radiation) [6]. The models were developed and validated according to The Organization for Economic Co-operation and Development (OECD) principles for the validation of QSAR models [7].

First, the most suitable models had to be selected for the prediction of the antioxidant activity [6]. Since the average of predictions from several models for the prediction of the same activity (also called consensus modeling) offers superior performances in comparison to the use of single models [69], we decided to employ all the suitable models. Two QSAR models (the MLR Fenton model and the MLR UV model) were discarded due to the lack of confidence in their performances, based on poor validation parameters [70], while the remaining seven models were employed for the prediction of activity of novel compounds, selected by 2D substructure search and the pharmacophore virtual screening.

#### 4.1.2. 2D substructure search

**Fig 10:**
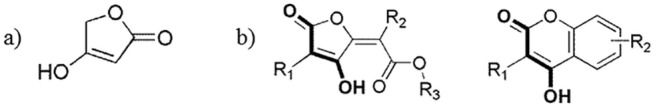
a) The structure of tetronic acid; b) the vinylogous acid fragment (bolded), common to the pulvinic acid and coumarine derivatives.

First, the eMolecules database was screened for derivatives of tetronic acid (Fig 10), which is five-membered ring fragment of the pulvinic acid containing a vinylogous acid fragment, presumed to be the bearer of the antioxidant activity of such compounds [5]. Only 29 hits were obtained when screening for compounds containing the tetronic acid fragment; since we were not satisfied with the number of obtained hits, the search was broadened to all compounds containing the vinylogous acid fragment (Fig 10). About 4.000 hits were obtained in this manner, which were further refined. Since the substructure search for the vinylogous acid fragment resulted in structurally very diverse hits, the main emphasis in the first step of the refinement was put on the similarity of selected compounds with the reference compounds used for the QSAR models development [6], while in the second step some compounds were removed due to their potentially problematic structural features (such as epoxides, azo compounds, etc.) Finally, a pool of 239 compounds was selected (provided in the Supporting Information as S1 File).

#### 4.1.3. Pharmacophore modeling

**Fig 11:**
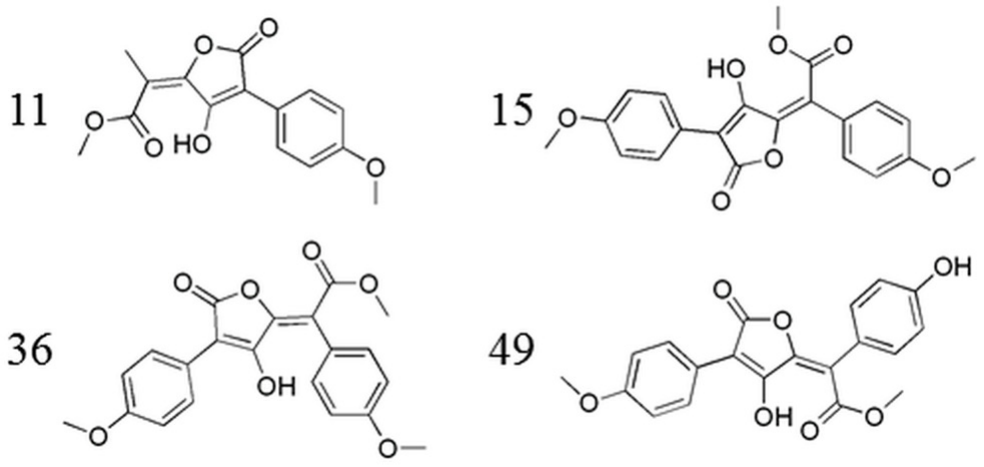
Four active pulvinic acid derivatives (IDs: 11, 15, 36, 49 [6]), selected for the construction of the pharmacophore model.

A ligand-based pharmacophore model was derived using LigandScout software [71], on the basis of four selected active pulvinic acid derivatives (IDs: 11, 15, 36, 49 [6]) (Fig 11). Several unique conformations were first calculated for each structure by applying LigandScout conformer generator coupled to the OMEGA software [72] with the following settings: maximum number of output conformers per molecule = 25; RMS threshold to duplicate conformers = 0.8 Å; maximum number of generated conformers per molecule = 30000; maximum number of intermediate conformers per molecule = 4000; maximum search time = 30 seconds; energy window = 10 kcal/mol. The generated conformers were dynamically aligned, and 10 merged feature pharmacophore models were produced. These were assessed using a scoring function combining the pharmacophore fit and the atom shape overlay, and the model with the highest score of 0.9010 was selected for further use.

**Fig 12:**
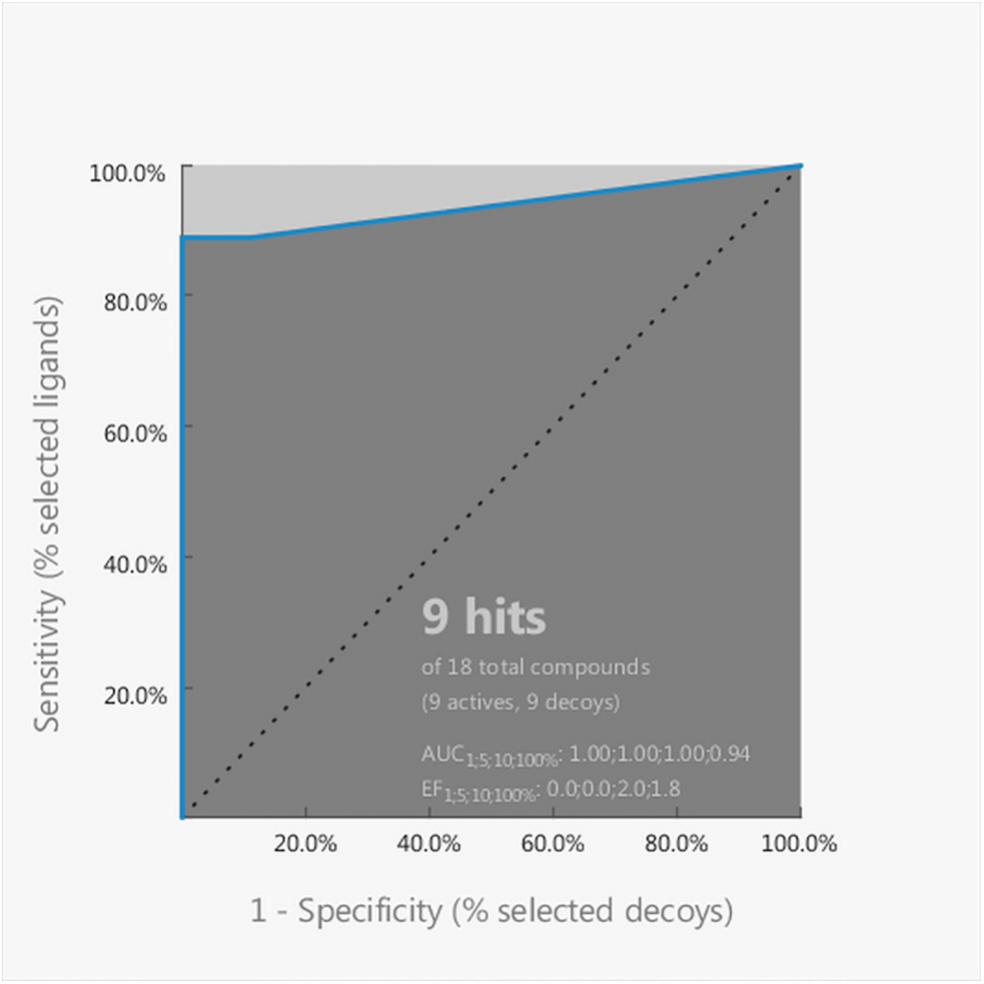
The ROC curve, obtained from the Plot ROC curve tool in the LigandScout screening module, together with the corresponding output values of AUC and EF.

The obtained pharmacophore model was successfully validated utilizing the Plot ROC curve tool in the LigandScout screening module, which performs the screening a series of known active and inactive compounds, constructs a receiver operating characteristic (ROC) curve [73,74] and outputs the area under the ROC curve (AUC) and the enrichment factor. The active and inactive pulvinic acid derivatives were selected on the basis of their experimentally determined antioxidant activity in the thymidine protection assay under the influence of different oxidative stressors [6]; the compounds that were on average among the most active in all the oxidative conditions were selected as active, while the compounds that were on average the least active in all the conditions were selected as inactive. The sets of active and inactive compound contained nine molecules each, and the set of actives included also the four compounds that were used for the development of the pharmacophore model; the structures of the compounds used for the pharmacophore model validation are provided in the S2 Table of the Supporting Information. The model successfully identified all 9 active compounds, while 8 of the decoys were determined as inactive and only 1 was misclassified as active. The models ability to discriminate active from inactive compounds is reflected by the AUC value, where the maximal possible value is 1; the AUC value obtained at 100% of the collection of active and inactive compounds had a satisfactory value of 0.94 (Fig 12). Another output parameter was the enrichment factor (EF), which reflects the capability of a screening tool to detect active compounds in comparison to a random selection [75]; the value should be always greater than 1, and our model produced a value of 1.8 at 100% of the collection of active and inactive compounds.

After the validation, the pharmacophore model was employed for the virtual screening of about two million commercially available compounds from the vendors Vitas-M and ChemDiv. From the obtained virtual hits, only the ones that fulfilled all of the derived pharmacophore constraints were selected, resulting in a pool of around 1000 virtual hit compounds, which were then all visually inspected to remove compounds with potentially problematic structural features, and to put emphasis on compounds which possess moieties dissimilar to chemical class of pulvinic acids with the potential antioxidant capacity. Finally, a total of 271 hits were obtained (provided in the Supporting Information as S2 File).

### 4.2. Materials

The novel compounds **1–9** were purchased from Vitas-M, **10** and **11** from Key Organics, **12–14** from ChemDiv, **15** from MolMall, **16** from MolPort, **17** from ChemBridge, and **18–21** from Sigma Aldrich, while the standard antioxidants aesculin, ascorbic acid, curcumin, scopoletin, Trolox and gallic acid were purchased from TCI Europe, resveratrol from ChromaDex, and quercetin from Sigma Aldrich. The structures of the novel compounds and standard antioxidants are presented in Fig 4.

2,2-diphenyl-1-picrylhydrazyl (DPPH), 2-deoxyribose, β-carotene, linoleic acid, lipoxidase from *Glycine max*, trypan blue solution, gentamicin, phorbol 12-myristate 13-acetate (PMA) and 2',7'-dichlorofluorescin diacetate (DCFH-DA) were all purchased from Sigma-Aldrich. Sterile dimethyl sulfoxide (DMSO) was obtained from WAK-Chemie Medical GmbH, fetal bovine serum (FBS), glutamine (GlutaMAX) and Dulbecco’s Phosphate-Buffered Saline (DPBS) from Gibco, Ficoll from Cedarlane, H_2_O_2_ from Merck, and RPMI 1640 medium from PAA Laboratories GmbH. All other chemicals (EDTA, FeCl_3_, methanol, tween 20, chloroform, thiobarbituric acid, trichloroacetic acid, NaOH, KH_2_PO_4_, and K_2_HPO_4_) were of reagent grades and obtained from the local chemical vendors in Slovenia.

### 4.3. Experimental methods

#### 4.3.1. DPPH assay

A solution of DPPH in methanol with a concentration of 140 μM was prepared daily. The evaluated compounds were also dissolved in methanol, directly before performing each experiment.

First, the steady state absorbance of DPPH was measured for each evaluated compound by mixing 900 μL of 140 μM DPPH solution and 900 μL of the sample in plastic cuvettes, which were then sealed with a paraffin film and stored in the dark. After the designated incubation time the absorbance at 516 nm was measured. Since the reaction kinetics varies widely among different substrates, [76] suggests to monitor the reaction until a plateau in absorbance is reached. Since some compounds were not able to reach the steady state within 60 minutes, the incubation time for such compounds was prolonged to 6 hours; however, since the steady state still had not been reached, the incubation time was further prolonged to 24 hours. All the compounds were evaluated in triplicates, at four different sample concentrations.

The percentage of the remaining DPPH in the steady state was calculated by using the following formula:

$$\%\;{DPPH}_{remaining}=\frac{A_{f}}{A_{0}}\times 100$$ (3)

where A_0_ corresponds to the initial DPPH absorbance and A_f_ represents the DPPH absorbance in the steady state.

The activities were expressed with the half maximal effective concentration (EC_50_) values, representing the concentration of the antioxidant that reduces 50% of the initial DPPH. The percentage of the remaining DPPH was plotted versus the logarithm of the antioxidant concentration, and a non-linear five-parameter asymmetrical regression was performed using the software GraphPad Prism^®^ version 6 (San Diego, CA) to calculate EC_50_, as suggested by [77].

The kinetic behavior of the compounds was also evaluated by calculating the second-order rate constants for the reaction with DPPH. Usually this is done by performing the experiments under pseudo first-order conditions, where the concentration of one reactant is significantly higher than that of the other. When the antioxidant is used in excess, the DPPH absorbance may decrease too fast for accurate measurements using conventional UV-spectroscopy, so we decided to use the excess of DPPH over the antioxidant, with the exception of aesculin and scopoletin, where higher concentrations of the antioxidant were needed to obtain a measurable effect.

Again, each compound was evaluated at four different sample concentrations, in triplicates. From the measured absorbances the corresponding concentrations of DPPH were calculated by using the equation, obtained from a calibration curve by Brand-Williams et al. [78]:

$$[DPPH]=\frac{A+2,58\times 10^{-3}}{12509}$$ (4)

where A stands for the measured absorbance and the concentration of DPPH is expressed in mol/L.

On the basis of these values pseudo first-order rate constants (*k*_obs_) were calculated. The type of calculation used depended on which of the reagents was used in excess. In majority of the cases, where [DPPH] > [antioxidant], *k*_obs_ were calculated by the following equation [79]:

$$\left[DPPH]={\left[DPPH\right]}_{0}({\left[\mathrm{antioxidant}\right]}_{0}\times e^{-k_{obs}t}\right)$$ (5)

In the case of aesculin and scopoletin, which had to be used at higher concentrations to produce a measurable response, *k*_obs_ were calculated using the following equation [26]:

$$[DPPH]={\left[DPPH\right]}_{0}\times e^{-k_{obs}t}$$ (6)

The calculated pseudo first-order rate constant values were plotted against the concentrations of the antioxidants used, and the slopes of these plots represent the second-order rate constants *k*_2_ of the tested compounds [26].

#### 4.3.2. 2-deoxyribose assay

The assay was carried out by a procedure described by Aruoma [80], with some modifications. 1.5 mL plastic tubes were used, containing the final concentrations of 9 mM phosphate buffer (pH = 7.4), 2.5 mM 2-deoxyribose (2-DR), 2.5 mM H_2_O_2_, 88.9 μM EDTA, 7.4 μM FeCl_3_, together with different concentrations of evaluated compounds. The production of hydroxyl radicals was initiated by the addition of ascorbic acid with the final concentration of 88.9 μM. Then the tubes were incubated in a water bath (37°C) for 1 hour. 460 μL of 1% (w/v) TBA in 50 mM NaOH and 460 μL of 2.8% (w/v) trichloroacetic acid were added, and the tubes were further incubated in a 90°C water bath for 20 minutes and were then allowed to cool. Finally, the absorbance at 532 nm was measured.

The antioxidant activities were expressed as percentages of the inhibition of 2-DR degradation, calculated by the following equation:

$$\%\;inhibition=\frac{A_{0}-A_{sample}}{A_{0}}\times 100$$ (7)

where A_0_ is the absorbance of the control sample and A_sample_ the absorbance of the sample under evaluation. The % inhibition values were plotted against the logarithm of the antioxidant concentration in order to calculate the half maximal inhibitory concentration (IC_50_) values, representing the concentration of a compound that inhibits 2-DR degradation by 50%. The calculation was performed in the GraphPad Prism software, using the non-linear asymmetric 5-parameter method. All compounds were evaluated using at least four different concentrations, and in triplicates.

#### 4.3.3. β-carotene bleaching assay (BCB)

A stock solution of β-carotene was prepared by dissolving 500 μL of β-carotene and 1.5 mL of Tween 20 in chloroform. Chloroform was evaporated and the stock solution was refrigerated at -20°C. Potassium phosphate buffer was prepared by dissolving 6.8 g KH_2_PO_4_ and 2.0 g of NaOH in Mili-Q water and adjusting the pH to 7.0. Immediately before each experiment, a small quantity of the β-carotene stock was diluted with the buffer, until the measured absorbance of the solution at 460 nm was equal to 0.68. Linoleic acid (LA) solution was prepared by mixing 50 μL of LA with 500 μL of Tween 20 and then diluting to 10 mL using the pH 7 buffer, while the lipoxydase (LOX) was dissolved in the buffer to a concentration of 1 mg/mL. Evaluated compounds were dissolved in methanol.

First the control sample was measured: 1.5 mL of β-carotene working solution was mixed with 120 μL of LA and 100 μL of methanol in a 3 mL plastic cuvette. 120 μL of LOX was added to initiate the reaction, and the absorbance at 460 nm was monitored for 10 minutes. Next, each of the samples was evaluated in the same manner as the control, with the exception of 100 μL of pure methanol being replaced with 100 μL of the compound solution.

The activities of the compounds at each evaluated concentration were expressed as a percentage of the antioxidant activity, calculated by the following formula [81]:

$$\%\;AOA=\left[1-\frac{A_{s}^{0}-A_{s}^{10}}{A_{c}^{0}-A_{c}^{10}}\right]\times 100$$ (8)

A_s_^0^ and A_s_^10^ represent the absorbance of the sample at 0 min and at 10 min, respectively, while A_c_^0^ and A_c_^10^ correspond to the absorbance of the control at 0 min and at 10 min, respectively. The % AOA values were plotted against the logarithm of the antioxidant concentration, to calculate the EC_50_ value, which represents the concentration of the compound that effectively protects 50% of the initial β-carotene from degradation. The calculation was performed in the software GraphPad Prism, using the non-linear asymmetric 5-parameter method. All compounds were evaluated at four different concentrations and in triplicates.

#### 4.3.4. Cellular antioxidant activity (CAA)

The peripheral blood mononuclear cells (PBMC) were isolated from the blood of healthy donors, using aseptic techniques. Ficoll was used to separate PBMC from the blood by density gradient centrifugation, and the cultures of PBMC were then grown at 37°C and 5% of CO_2_.

The stock solutions of the evaluated antioxidants were prepared by dissolving them in dimethyl sulfoxide (DMSO) to a concentration of 25 mM (with the exception of compound **12**, which was only soluble at 12.5 mM), and then refrigerated at -20°C. Prior to each experiment, they were further diluted to the appropriate concentrations using the Dulbecco’s Phosphate-Buffered Saline (DPBS).

Next, the cytotoxicity of the evaluated compounds was determined, where the cells were incubated for 24 hours in the presence of a specified concentration of an antioxidant, after which the cell viability was checked by flow cytometry using propidium iodide as a marker of dead cells.

Finally the antioxidant activity was assessed; each compound was evaluated bellow its toxic concentration. 1 mL of the medium (RPMI 1640 and 10% fetal bovine serum) and 2.5 μL of the compounds stock solution were mixed in a plastic test tube and then transferred to a microwell plate, where about a million of PBMC were added to each well. The plate was incubated for 1 hour (37°C, 5% CO_2_), and then 1 μL of 20 mM 2′,7′-dichlorodihydrofluorescin diacetate (DCFH-DA) in DMSO was added. After another 30 minutes of incubation phorbol 12-myristate 13-acetate (PMA) was added to induce the production of reactive oxygen species (ROS). After another hour of incubation the content of each well was transferred into a centrifuge tube, 4 mL of DPBS were added, and the tubes were centrifuged to wash the cells and remove any excess dichlorofluorescein (DCF) on the cell surface. The supernatant was discarded, DPBS was added again and the centrifuge was repeated. The precipitate was dissolved in 400 μL of DPBS and transferred into flow cytometry test tubes to measure the fluorescence. The control sample was treated in the same manner; the only difference was no antioxidant was added.

The activities of compounds at a given concentration were expressed with a CAA value, calculated as followed [63]:

$$CAA\;value=100-\left(\frac{\int SA}{\int CA}\right)\times 100$$ (9)

where ∫SA represents the integrated area under the sample fluorescence and ∫CA the integrated area from the control curve. All compounds were evaluated at two different concentrations, and the measurements were performed in triplicates.

## Supporting Information

S1 File A sdf file of 239 compounds, selected from the 2D substructure search. (SDF)

S2 File A sdf file of 271 compounds, selected from the 2D substructure search. (SDF)

S1 Table The EC_50_/IC_50_ curves obtained from the performed experimental assays. (XLSX)

S2 Table Structures of active and inactive compounds (together with their ID values [6]), that were used for the validation of the derived ligand-based pharmacophore model. (DOCX)
