# Supplementary material for: In Silico Discovery of Novel Potent Antioxidants on the Basis of Pulvinic Acid and Coumarine Derivatives and Their Experimental Evaluation
Source: PLoS One. 2015 Oct 16;10(10):e0140602. doi: 10.1371/journal.pone.0140602 (PMC4608598; doi:10.1371/journal.pone.0140602)
Supplement: S2 Table — (DOCX) [file pone.0140602.s004.docx]

**S1 Table.** Structures of active and inactive compounds (together with their ID values [6]), that were used for the validation of the derived ligand-based pharmacophore model.

| Active compounds | Inactive compounds |
| --- | --- |
| 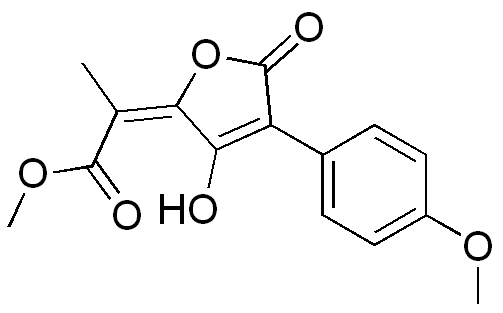ID: 11 | 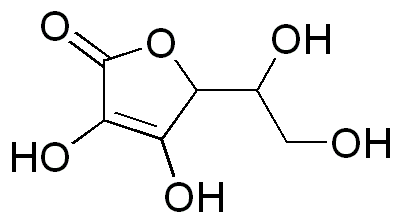ID: 30 |
| 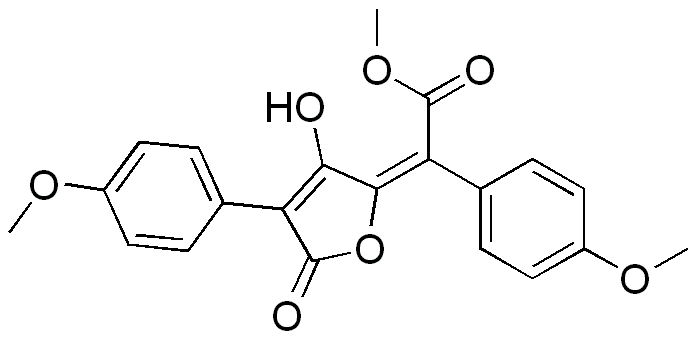ID: 15 | 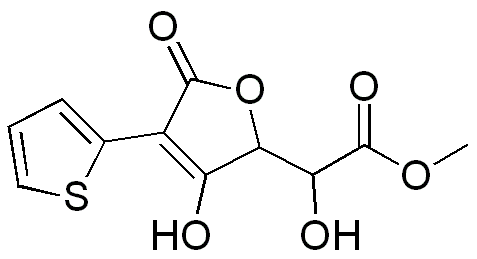ID: 62 |
| 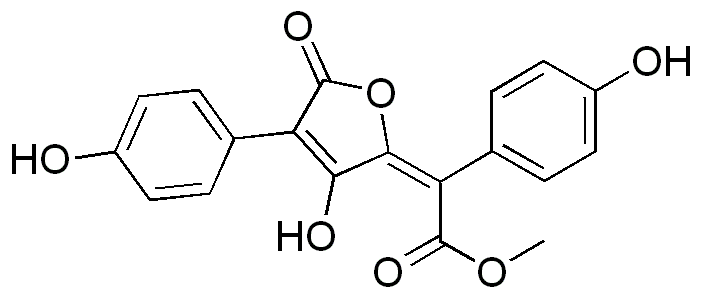ID: 33 | 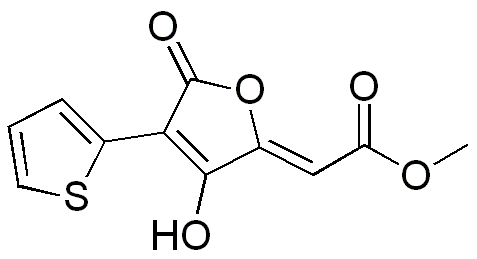ID: 63 |
| 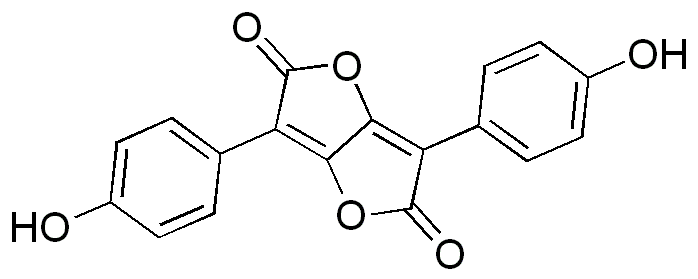ID: 35 | 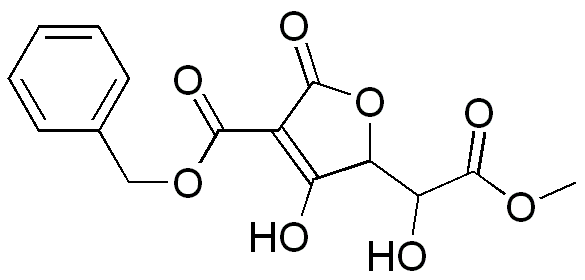ID: 66 |
| 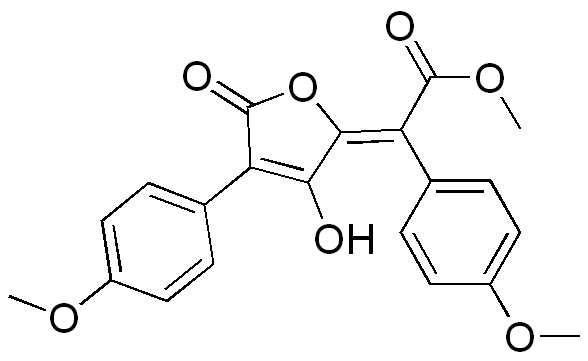ID: 36 | 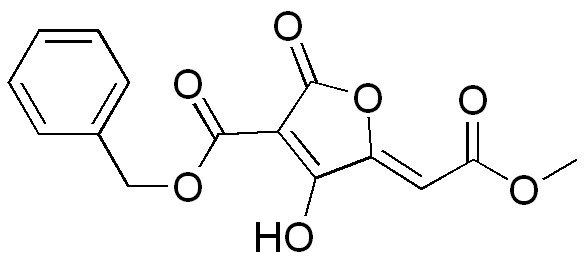ID: 67 |
| 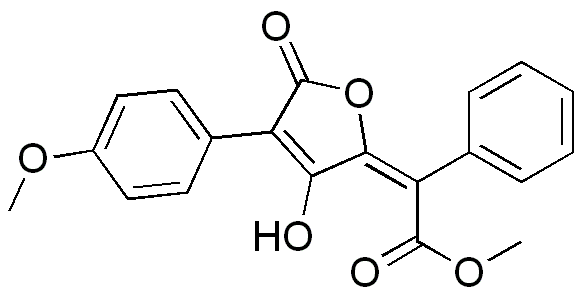ID: 40 | 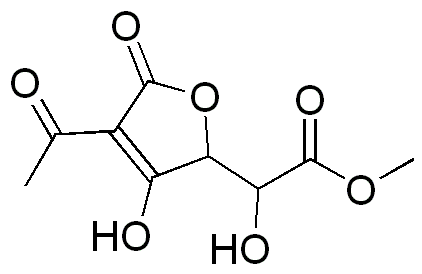ID: 69 |
| 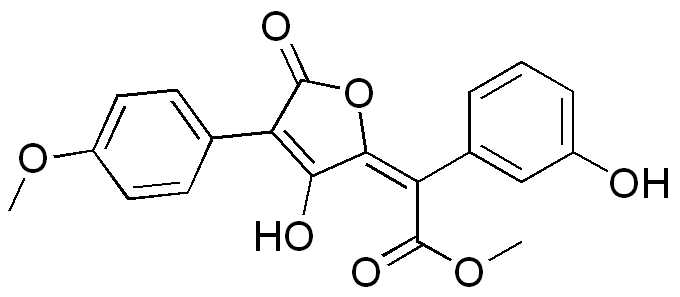ID: 41 | 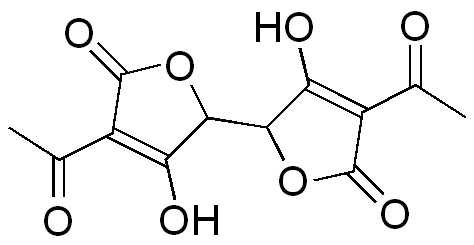ID: 70 |
| 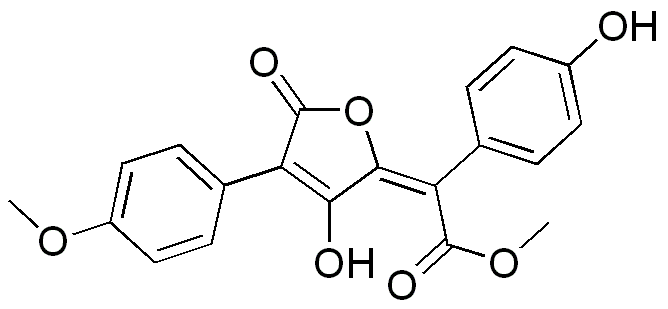ID: 49 | 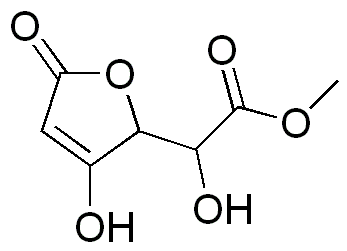ID: 71 |
| 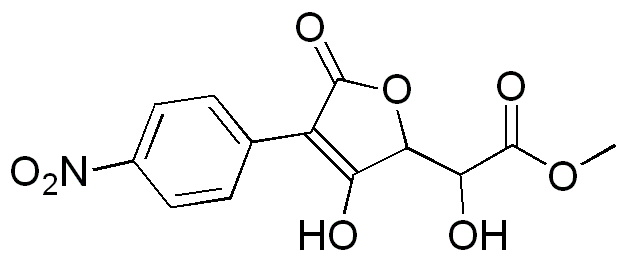ID:57 | 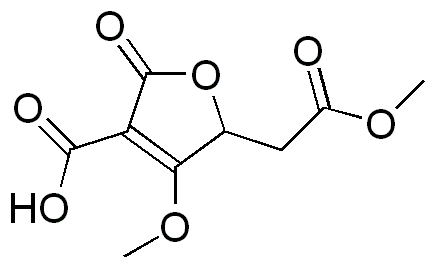ID:72 |
